# Hyperphosphorylation and Cleavage at D421 Enhance Tau Secretion

**DOI:** 10.1371/journal.pone.0036873

**Published:** 2012-05-15

**Authors:** Vanessa Plouffe, Nguyen-Vi Mohamed, Jessica Rivest-McGraw, Johanne Bertrand, Michel Lauzon, Nicole Leclerc

**Affiliations:** Département de pathologie et biologie cellulaire, Université de Montréal, Montréal, Québec, Canada; National Institutes of Health, United States of America

## Abstract

It is well established that tau pathology propagates in a predictable manner in Alzheimer’s disease (AD). Moreover, tau accumulates in the cerebrospinal fluid (CSF) of AD’s patients. The mechanisms underlying the propagation of tau pathology and its accumulation in the CSF remain to be elucidated. Recent studies have reported that human tau was secreted by neurons and non-neuronal cells when it was overexpressed indicating that tau secretion could contribute to the spreading of tau pathology in the brain and could lead to its accumulation in the CSF. In the present study, we showed that the overexpression of human tau resulted in its secretion by Hela cells. The main form of tau secreted by these cells was cleaved at the C-terminal. Surprisingly, secreted tau was dephosphorylated at several sites in comparison to intracellular tau which presented a strong immunoreactivity to all phospho-dependent antibodies tested. Our data also revealed that phosphorylation and cleavage of tau favored its secretion by Hela cells. Indeed, the mimicking of phosphorylation at 12 sites known to be phosphorylated in AD enhanced tau secretion. A mutant form of tau truncated at D421, the preferential cleavage site of caspase-3, was also significantly more secreted than wild-type tau. Taken together, our results indicate that hyperphosphorylation and cleavage of tau by favoring its secretion could contribute to the propagation of tau pathology in the brain and its accumulation in the CSF.

## Introduction

The microtubule-associated protein tau that is normally enriched in the axon becomes hyperphosphorylated and accumulates in the somato-dendritic compartment in several neurodegenerative diseases named tauopathies that are characterized by dementia [1], [2]. In these diseases that include AD, tau aggregates in insoluble filaments that form lesions called neurofibrillary tangles (NFTs) [3]. The appearence of these lesions in a predictable manner in the brain correlates with the degree of cognitive deficits [4], [5], [6], [7]. Moreover, the amount of tau found in CSF increases during progression of AD [8]. It remains unclear how tau pathology propagates in the brain and how tau reaches the CSF. Recent studies have reported that the secretion of tau could contribute to both of these events. In vitro, tau was shown to be secreted by M1C, NB2a/d1, COS-7 and KEK-293 cells [9], [10], [11]. When human tau cDNA was microinjected in central lamprey neurons, human tau could transfer from one neuron to another indicating that secreted tau could be involved in the propagation of the disease in vivo [10]. However, it was unclear whether this phenomenon was specific to this model until two recent studies demonstrating the trans-synaptic propagation of tau pathology in a mouse model [12], [13]. In this model, where human tau overexpression was restricted in the entorhinal cortex, the first region to be affected in AD, the spreading of tau pathology was observed along synaptically connected circuits. From these studies, one can conclude that the secretion of tau at the synapse might be involved in the propagation of tau pathology in mouse brain. Tau secretion could also result in the increase of tau in the CSF as reported in a study showing that the increased amount of tau in the CSF could not be linked to neurodegeneration in tau transgenic mouse models [14].

In AD, tau is phosphorylated at more than 40 sites compared to 9 sites in normal patients [15]. Until now, only few phosphorylation sites were examined in CSF. In several studies, both the amount of total tau and phosphorylated tau (ptau) were measured in the CSF [8]. Indeed, the ratio of ptau/total tau was shown to be more accurate in distinguishing Alzheimer’s patients from controls than the mere measure of total tau. The phosphorylation of threonine 181 (T181) is extensively used for measuring tau in the CSF [8]. In AD and in mildly cognitive impaired patients, the phosphorylation of T181 is significantly higher than in normal patients whereas it is decreased in patients presenting a fronto-temporal dementia (FTD) [16], [17]. Moreover, phopshorylation of T181 was used to differentiate AD from dementia with Lewy bodies (DLB) [18]. The phosphorylation of T231 was also increased in CSF tau obtained from AD patients [19], [20], [21], [22], [23]. However, some studies reported that the phosphorylation of T231 decreased with the progression of AD [24]. The phosphorylation of T231 is lower in FTD than in AD [18]. The above observations revealed that the distinct pattern of tau phosphorylation could be used to discriminate between tauopathies. The phosphorylation of other sites such as S199, S202 and T205 (epitope recognized by the phospho-tau antibody AT8) and S396 and S404 (epitope recognized by the phospho-tau antibody PHF-1) were less examined and their phosphorylation in CSF remains controversial [25]. The low amount of tau in the CSF has been a limiting factor in characterizing its phosphorylation state. So far, the increase of tau in the CSF was attributed to neuronal cell death. A recent study reported that intracellular tau released in the culture medium upon cell death was dephosphorylated [26]. It remains to be determined whether tau found in the CSF has a phosphorylation pattern similar to that of intraneuronal tau.

Tau found in the CSF of AD and progressive supranuclear palsy (PSP) patients is cleaved [27], [28], [29]. The main cleavage seems to occur at the C-terminal. It is still unclear whether tau is cleaved before it is released in the CSF. A study reported that when full-length tau was added to the CSF, it did not get cleaved indicating that the cleavage of tau took place before its release in the CSF [27]. Interestingly, CSF-tau obtained from tau transgenic mice was also cleaved at the C-terminal [14]. A recent study reported that tau secreted by M1C and NB2a/d1 cell lines was cleaved at the C-terminal in a pattern reminiscent to tau found in the CSF [9], [30]. All together, the above observations revealed that tau cleaved at the C-terminal is preferentially released in the CSF.

Until now, no study has examined whether phosphorylation and cleavage of tau favor its secretion. In the present study, we showed that the overexpression of human tau resulted in its secretion by Hela cells. Interestingly, secreted tau was dephosphorylated at several sites in comparison to intracellular tau and that only tau cleaved at the C-terminal was found in the medium. Our data also revealed that hyperphosphorylation and cleavage of tau favored its secretion by Hela cells. Therefore, hyperphosphorylation and cleavage enhancing the secretion of tau in AD brain could contribute to the propagation of its pathology in the brain and to its accumulation in the CSF. From the present results showing that secreted tau was dephosphorylated and the previous study reporting that tau released in the culture medium upon cell death was dephosphorylated, one can also speculate that tau found in the CSF would be dephosphorylated.

## Materials and Methods

### Cell Culture and Transfection

Hela cells (ATCC, Manassas, VA, USA) were cultured in DMEM (Invitrogen, Burlington, ON, Canada) supplemented with 10% foetal bovine serum (Hyclone, Logan, UT) and 2 mM L-glutamine (Sigma, Oakville, ON, Canada) at 37°C in a humidified 5% CO_2_ incubator. For transfection, Hela cells were plated at a density of 1.1 × 10^6^ cells in 60-mm Petri dishes and grown overnight to 80% confluency. Lipofectamine 2000 (Invitrogen) was used to transfect Hela cells with the expression vector (pEGFP-C1 from Clonetech) containing either wild-type human 4R tau (wild-type htau) or human 4R tau mutant fused at the C-terminus of a Green Fluorescent Protein (GFP) tag or with the pRc/CMV vector containing wild-type human tau fused at the C-terminus of a Flag tag (kindly provided by Dr. Gloria Lee, University of Iowa, Iowa, IA). Briefly, for each petri, 8 µg of plasmid DNA was mixed with 500 µl of Opti-MEM medium (Invitrogen), and 16 µl of Lipofectamine 2000 was mixed with 500 µl of Opti-MEM medium. Both mixtures were incubated for 5 min then mixed and left standing for 20 min. Then, 1 ml of the mixture was added to each petri. After an incubation of 4 hrs at 37°C, culture medium was replaced by 6 ml of fresh medium. The GFP-4Rtau construct was kindly provided by Dr. Ken Kosik (University of California, Santa Barbara, CA, USA; Lu and Kosik 2001), human 4R tau mutants, A12 and E12, containing 12 phosphorylation sites (S199, S202, T205, S214, T231, S235, S262, S356, S396, S400, S404 and S409) mutated in alanine and glutamate respectively, were modified from the GFP-4Rtau construct in our laboratory and tauΔ413–441 and tauΔ422–441 were generated from GFP-4Rtau construct by Mutagenex (Piscataway, NJ, USA). Two days after transfection or as mentioned in the text, culture medium was harvested and cells were lysed for immunoblotting.

### Preparation of Cell Lysate and Culture Medium Containing Tau

Two days after transfection, the culture medium was collected and centrifuged at 3000 RPM for 10 min at room temperature to remove cell debris. After the culture medium was collected, the cells were immediately washed twice with phosphate buffered saline (PBS) and once with PBS containing 0.5 M NaCl to detach proteins non-specifically attached at the cell surface [31]. The cells were then lysed in 6 ml of fresh culture medium supplemented with 0.1% Triton X-100 and protease inhibitor cocktail 1X (Complete EDTA-free from Roche Diagnostics, Indianapolis, IN, USA) and then incubated on ice for 10 min. The cell lysate was vortexed and then centrifugated at 3000 RPM for 10 min at room temperature.

### Isolation of Microvesicles/exosomes

Two days after transfection, the culture medium was collected. The isolation of microvesicles/exosomes was performed using differential centrifugation as described by Thery et al. [32]. Briefly, the culture medium was centrifuged at 300xg for 10 min, then at 2000xg for 10 min and at 10,000xg for 30 min at 4°C to remove cell debris. The microvesicles/exosomes were isolated by a centrifugation at 100,000xg for 120 min at 4°C. Microvesicles/exosomes were washed in PBS and centrifuged again at 100,000xg for 60 min at 4°C. The presence of tau in the pellet containing the microvesicles/exosomes was analyzed by western blotting.

### Immunoprecipitation

To analyze the phosphorylation pattern of secreted tau, tau was immunoprecipitated from the culture medium and the cell lysate. Magnetic beads coupled with anti-mouse antibodies (DYNAL Biotech, Dynabeads® M-280 Sheep anti-Mouse IgG) were washed in PBS and incubated O/N at 4°C with the following antibodies: 0.3 µg Tau-1, 0.1 µg HT7 and 6 µl CP13 (kindly provided by Dr. Peter Davies, Albert Einstein University, Bronx, NY, USA). The beads were then washed and incubated for 2 hrs at 4°C with 1.5 ml of the culture medium or the cell lysate. The complex bead-antibody-antigen was then washed, resuspended in 80 µl of sample buffer 1X and boiled for 5 min. Then, 40 µl of the samples were loaded per well and separated on 7.5% polyacrylamide gel. Immunoblotting was performed as described below.

### Immunoblotting

Equal amount of the culture medium and the cell lysate (20 or 40 µl) were loaded in each lane and electrophoresed on a 7.5% polyacrylamide gel. Following separation, proteins were electrophoretically transferred to a nitrocellulose membrane. The nitrocellulose stripes were incubated with the primary antibodies O/N at 4°C. They were then washed with Tris-buffered saline with 0.2% Tween-20 (Sigma) and incubated with the peroxidase-conjugated secondary antibodies (Jackson Immunoresearch Laboratories, Missisauga, ON, Canada). Membranes were again washed and revealed by chemiluminescence (Amersham Pharmacia Biotech, Quebec, Quebec, Canada). Many tau antibodies were used to visualize either the phosphorylation state or different epitopes of the protein (see Table 1). We also used a mouse monoclonal anti-tubulin (1∶2000) (clone DM1A from Sigma) to assess the cell death and a mouse monoclonal anti-GFP antibody (mix of clones 7.1 and 13.1) (1∶5000) (Roche Diagnostics).

**Table 1:** List of tau antibodies.

Antibody	Type	Epitope	WB dilution	Source
HT7	Mouse monoclonal	between a.a. 159–163	1∶500	Pierce Biotechnology, Rockford, IL, USA
K9JA	Rabbit polyclonal	a.a. 243–441	1∶20 000	DakoCytomation, Glostrup, Denmark
Tau1	Mouse monoclonal	dephosphorylated a.a. 195, 198, 199 and 202	n/a	Millipore, Billerica, MA, USA
Tau12	Mouse monoclonal	Between a.a. 9–18	1∶5000–1∶20 000	Abcam, Cambridge, MA, USA
Tau46	Mouse monoclonal	a.a. 428–441	1∶500	Abcam, Cambridge, MA, USA
AT270	Mouse monoclonal	pT181	1∶100	Pierce Biotechnology, Rockford, IL, USA
Phospho-S199	Rabbit polyclonal	pS199	1∶1000	Biosource-Invitrogen, Burlington, ON, Canada
CP13	Mouse monoclonal	pS202	1∶50	Kindly provided by Dr. Peter Davies, Albert Einstein College of Medicine, NY, USA
Phospho-T205	Rabbit polyclonal	pT205	1∶500	Biosource-Invitrogen, Burlington, ON, Canada
Phospho-T212	Rabbit polyclonal	pT212	1∶1000	Biosource-Invitrogen, Burlington, ON, Canada
Phospho-S214	Rabbit polyclonal	pS214	1∶100	Biosource-Invitrogen, Burlington, ON, Canada
Phospho-T217	Rabbit polyclonal	pT217	1∶1000	Biosource-Invitrogen, Burlington, ON, Canada
Phospho-S262	Rabbit polyclonal	pS262	1∶1000	Signalway antibody, Pearland, TX, USA
Phospho-S409	Rabbit polyclonal	pS409	1∶100	Biosource-Invitrogen, Burlington, ON, Canada
Phospho-S422	Rabbit polyclonal	pS422	1∶1000	Biosource-Invitrogen, Burlington, ON, Canada
AT180	Mouse monoclonal	pT231/pS235	1∶100	Pierce Biotechnology, Rockford, IL, USA
PHF-1	Mouse monoclonal	pS396/pS404	1∶100	Kindly provided by Dr. Peter Davies, Albert Einstein College of Medicine, NY, USA

### Treatment with Brefeldin A, Caspase-3 Inhibitor and Low Temperature Assays

Two days after transfection, the culture medium was changed for fresh medium and the cells were treated for 4∶30 hrs with brefeldin A (BFA) (Sigma) at a concentration of 5 µg/ml, diluted in 0.05% of dimethyl sulfoxide (DMSO) (Sigma). Control cells were treated with 0.05% DMSO. Cells were also treated with the caspase-3 inhibitor Z-DEVD-FMK (TOCRIS, Minneapolis, MN, USA). Twenty-four hrs after transfection, the culture medium was changed for fresh medium and the cells were treated with 20 µM of the caspase-3 inhibitor or 20 µM of DMSO for 24 hrs.

Alternatively, cells were subjected to changes in temperature. To do so, cells were plated in 25 cm^2^ flasks instead of 60-mm petri dishes. The transfection was performed as described above. Two days after transfection, culture medium was changed for fresh medium and the cells were incubated at 18 or 4°C for 6 hrs. Control cells were incubated at 37°C. The cell lysate and the culture medium containing tau were prepared as described above.

### Quantification of Cell Death

Cell death was assessed by the measurement of the LDH activity in the culture medium and the cell lysate using the LDH cytotoxicity assay kit from Cayman Chemical Company (Ann Arbor, MI, USA), according to the manufacturer’s instructions. The cell death percentage was evaluated by the LDH activity in the culture medium (M) and the cell lysate (L) using the following formula: LDH Activity in M/total LDH Activity (Activity in M + L). Triplicates were performed for each sample. The LDH content in the samples was measured with a BIO-TEK Elx800 plate reader. The percentages of cell death are presented as the mean ± standard error of the mean (SEM).

Cell death was also evaluated by trypan blue exclusion method. Briefly, Hela cells were cultured on glass coverslip. Two days after transfection, cells were washed twice with PBS and then incubated in 0.2% trypan blue (Sigma) diluted in PBS for 4 min at room temperature (RT). Cells were then washed once with PBS and fixed in 4% paraformaldehyde in PBS for 5 min at 4°C and 10 min at RT. Cultures were kept in PBS until they were observed by light microscopy. The number of blue cells (dead cells) and total cells were counted on ten different fields and the cell death percentage was evaluated by the ratio of blue cells on total cells.

### Quantification of the Signal Detected with Tau Antibodies by Densitometry

Films were scanned with an EPSON PERFECTION 1240U scanner and transparency module EPSON EU-33, using Adobe Photoshop version 7.0 software. Image J software version 1.38X from the National Institute of Health was used to quantify the intensity of the band obtained with the different antibodies in the culture medium and the cell lysate prepared from Hela cells overexpressing either wild-type tau or mutated tau. To compare the secretion levels of wild-type tau with that of tau mutants, A12, E12, tauΔ413–441 and tauΔ422–441, the immunoblots were stained with the anti-tau antibody Tau12 that recognizes phosphorylated and non-phosphorylated tau and then the intensity of bands was measured by densitometry to calculate the ratio of the signal in the culture medium and the cell lysate. To compare the secretion levels of wild-type tau with or without the caspase-3 inhibitor, the immunoblots were stained with the anti-tau antibody Tau12 and then the intensity of bands was measured by densitometry to calculate the ratio of the signal in the culture medium and the cell lysate.

### Statistical Analysis

Statistical significance was evaluated with a one-way analysis of variance (ANOVA) followed by Dunnett multiple comparisons test against tau mutant E12 for the set of experiments on the effects of phosphorylation on tau secretion and against wild-type tau for the set of experiments on the effects of the C-terminal cleavage on tau secretion. The effect of caspase-3 inhibition on tau secretion was analyzed by a Paired t test. The statistical analysis was performed using the GraphPad InStat 3 software and *p*<0.05 was considered significant.

## Results

### Human Tau is Secreted by Hela Cells

**Figure 1:**
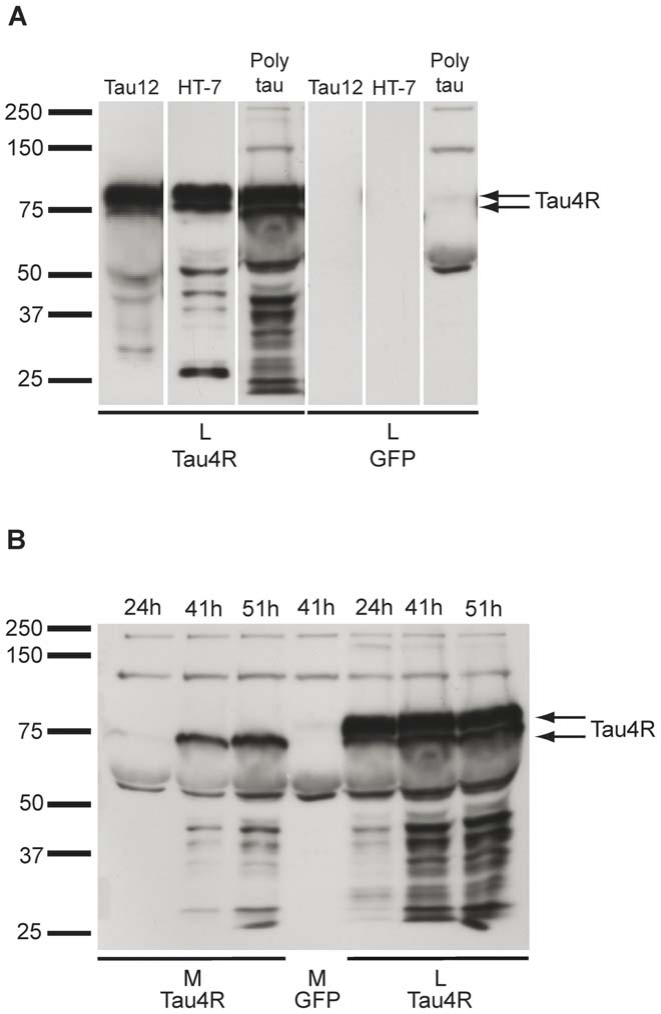
Overexpressed human tau in Hela cells is found in the culture medium. (A) In the cell lysate (L) prepared from Hela cells overexpressing human tau fused to the GFP tag, a tau- positive band just above 75 kDa and a band at 75 kDa corresponding to full-length and cleaved tau, respectively, were observed with the anti-tau antibodies Tau12, HT7 and a polyclonal antibody (arrows). No signal was detected with the anti-tau antibodies in L prepared from Hela cells transfected with the empty GFP-vector except for three non-specific bands detected with the polyclonal anti-tau antibody. Tau-positive bands migrating between 37 and 50 kDa and 25 and 37 kDa were also observed with the anti-tau antibodies. (B) The tau-positive band found at 75 kDa in L was also detected in the culture medium (M) and it increased with time as noted at 24, 41 and 51 hrs after transfection. No tau-positive bands were noted in M collected from cells transfected with the empty GFP vector except one non-specific band at ∼50 kDa detected with the polyclonal anti-tau antibody.

Human tau fused to the GFP tag (GFP-tau) was overexpressed in Hela cells. In the cell lysate at 48 hrs after transfection, a tau-postive band revealed by the anti-tau antibody Tau12 was found just above 75 kDa as expected when tau is fused to GFP (Figure 1A). A Tau12- positive band at 75 kDa as well as lower molecular weight bands between 37 and 50 kDa and 25 and 37 kDa were consistently observed but at a significantly lower intensity than the band of full-length tau indicating that overexpressed human tau was cleaved in Hela cells. A similar pattern of tau-positive bands was observed with the anti-tau antibody, HT7, and a polyclonal anti-tau antibody (poly anti-tau). No tau-positive band was detected with the anti-tau antibodies Tau12 and HT7 but three non-specific bands were detected with the polyclonal anti-tau antibody when cell lysates were prepared from Hela cells transfected with the empty GFP vector (Figure 1A). Surprisingly, the tau-positive band found at 75 kDa in the cell lysate was also present in the culture medium and became more abundant with time as noted at 24, 41 and 51 hrs after transfection (Figure 1B). However, full-length tau was consistently absent from the culture medium. Tau-positive bands were also noted between 37 and 50 kDa and 25 and 37 kDa in the culture medium as observed in the cell lysate. The fact that tau-positive bands detected in the medium were also noted in the cell lysate could indicate that some extracellular tau remained attached to the cells after washing them before lysis. To verify this possibility, cells were washed with PBS containing 0.5 M NaCl, a solution used to remove non-specific binding of proteins at the surface of cultured cells [31]. Under this washing condition, the tau-positive band at 75 kDa was still present in the cell lysate indicating that it could correspond to an intracellular pool of cleaved tau.

**Figure 2:**
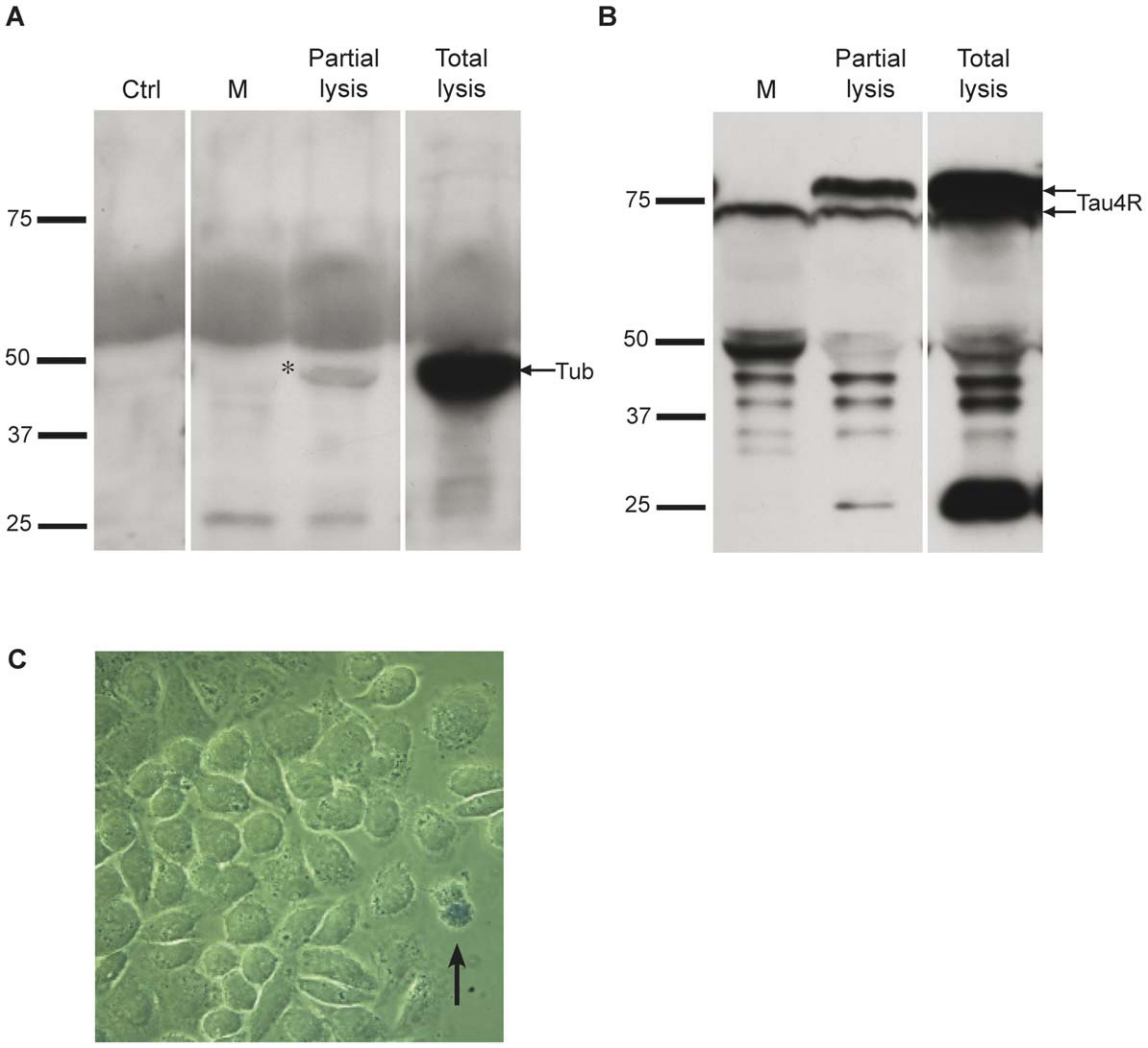
Overexpressed human tau is secreted by Hela cells. (A) No tubulin was noted in M before and after overexpression of human tau whereas tubulin staining was detected in the cell lysate (Total lysis) prepared in 6 ml of lysis buffer for comparison with the 6 ml of medium used to maintain Hela cells after transfection (arrow). In M collected from Hela cells overexpressing tau that were partially lysed (Partial Lysis) for few seconds in a solution of 0.01% Triton X-100 to induce some damage at the plasma membrane, tubulin staining became detectable (asterisk). (B) Cleaved tau was detected in M and L (lower arrow) whereas full-length tau was only detected in L (upper arrow in Total lysis). Full-length tau became detectable in M when Hela cells were partially lysed (Partial lysis) with a solution of 0.01% Triton X-100 (upper arrow). (C) Hela cells overexpressing human tau were stained with Trypan blue before being fixed to evaluate the percentage of cell death. Blue cells (arrow) corresponded to dead cells that had taken up Trypan blue.

**Figure 3:**
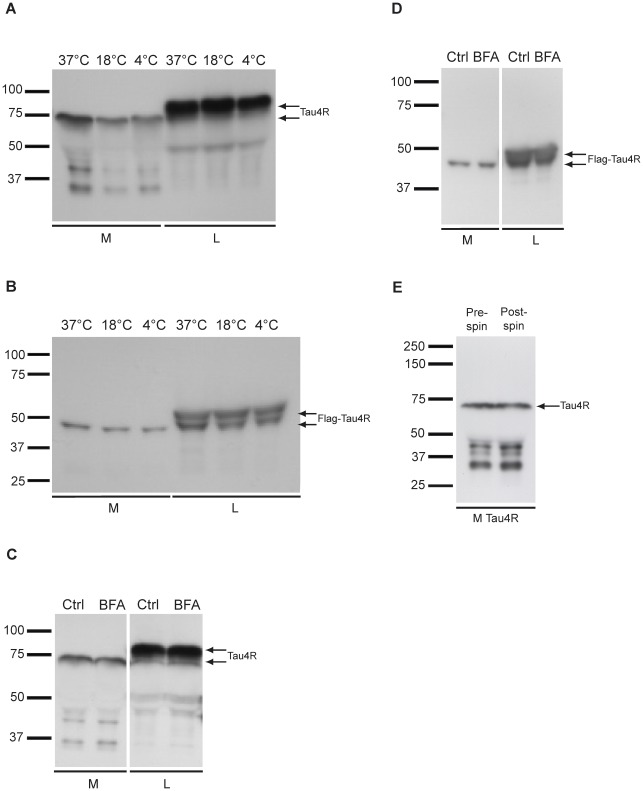
Secretion of human tau is reduced at low temperature and is not prevented by BFA treatment. (A) The amount of GFP-tau in M (lower arrow) was reduced at low temperature (18°C and 4°C) whereas the expression of tau was not affected (upper arrow). (B) Human tau fused to a Flag tag was also secreted by Hela cells. Secreted Flag-tau was cleaved as noted for GFP-tau (upper and lower arrows). The secretion of Flag-tau (lower arrow) but not its expression (upper arrow) was also impaired at low temperature. (C and D) The secretion of both GFP-tau and Flag-tau was not affected by BFA. Control cells (Ctrl) were treated with DMSO, the vehicle of BFA. BFA treatment was tested at least in three sets of experiments. E) The amount of wild-type tau in M was not decreased after ultracentrifugation to remove microvesicles/exosomes.

We next examined whether tau found in the medium was released by Hela cells either through cell death or secretion. To demonstrate that the presence of tau in the culture medium was not caused by membrane leakage from dying cells but rather by an active process of secretion, three approaches were used. First, the presence of a cytosolic protein such as tubulin in the culture medium from control and cells overexpressing tau was analyzed (Figure 2A). No tubulin was noted in the culture medium before and after overexpression of human tau consistent with the fact that no cell lysis was induced by the overexpression of human tau in Hela cells. In the cell lysate of tau transfected cells prepared in 6 ml of lysis buffer for comparison with the 6 ml of medium used to maintain Hela cells after transfection, tubulin staining was detected (Figure 2A). To further confirm that the presence of tau in the medium was caused by its secretion and not cell lysis, Hela cells overexpressing tau were partially lysed for few seconds in a solution of 0.01% Triton X-100 to induce some damage at the plasma membrane. In this condition, tubulin and full-length tau were detected in the medium confirming that full-length tau and tubulin would be found in the culture medium if Hela cells had been damaged by tau overexpression (Figure 2A and B). Second, cell death was evaluated by the trypan blue exclusion method and by the lactate dehydrogenase (LDH) activity measurement in the medium (Figure 2C) [33], [34]. From the trypan blue staining, it was possible to conclude that the presence of tau in the medium was not caused by cell death since an important amount of tau was found in the medium even when cell death was evaluated to be less than 1%. The LDH activity in the culture medium was measured for each set of experiments and allowed us to determine that the cell death percentage of Hela cells overexpressing tau varied from 0% to 5% in most experiments. From the trypan blue staining and LDH activity, no correlation could be established between the percentage of cell death and the amount of tau in the medium confirming the secretion of tau by Hela cells. Third, to demonstrate that tau was secreted by an active process by Hela cells, the secretion of tau was examined when the cells were incubated at low temperature, a condition known to decrease secretion by exocytosis [34], [35]. The amount of tau in the medium was significantly reduced at low temperature although no difference was noted between 18°C and 4°C (Figure 3A). The percentage of cell death was 0.35±0.35, 3.2±1.1 and 2.64±1.87 at 37°C, 18°C and 4°C respectively. To eliminate the possibility that the GFP tag which can be secreted when misfolded could contribute to tau secretion, the secretion of human tau fused to a Flag tag was examined in Hela cells [36]. As noted for GFP-tau, Flag-tau was found in the medium and its secretion was also impaired at low temperature (Figure 3B). The percentage of cell death was 0, 3.67±0.30 and 3.32±0.54 at 37°C, 18°C and 4°C respectively. For both GFP-tau and Flag-tau, an increase of cell death was noted at low temperature whereas tau secretion was decreased showing that the presence of tau in the culture medium was not imputable to cell lysis.

We next examined whether tau was secreted through the conventional pathway by treating the cells with brefeldin A (BFA), a drug known to inhibit this secretory pathway [37]. BFA did not prevent the secretion of GFP-tau and Flag-tau by Hela cells indicating that their secretion occurs through a non-conventional pathway (percentage of cell death: 0% for control and BFA treated cells)(Figure 3C and D).

In previous studies, it was shown that secreted tau was found in microvesicles and exosomes [11], [30]. We examined whether tau secreted by Hela cells was included in vesicles. Medium containing tau was centrifuged to isolate microvesicles/exosomes as described by Thery et al. [32]. The presence of tau in the supernatant and pellet was examined by western blotting. The amount of tau present in the supernatant was similar to that found in the medium that has not been centrifuged indicating that the major portion of tau secreted by Hela cells was not included in microvesicles/exosomes (Figure 3E). However, tau could be detected in the pellet when it was resuspended in a small volume revealing that a small pool of secreted tau was found in microvesicles/exosomes (data not shown).

### Human Tau Secreted by Hela Cells is Cleaved at the C-terminal

**Figure 4:**
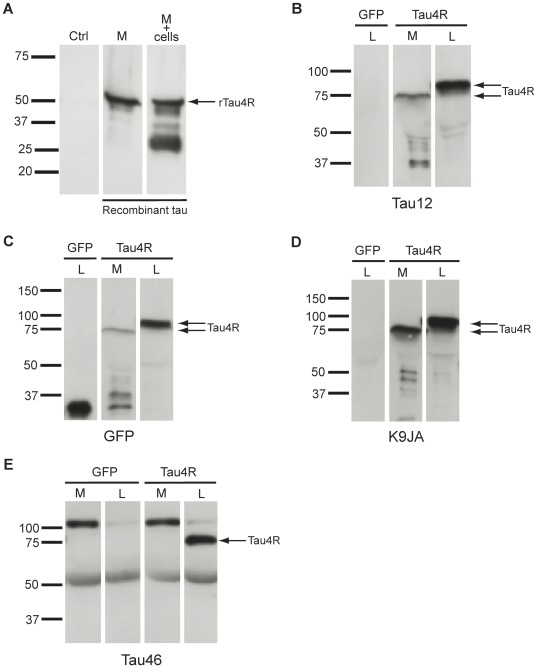
Secreted tau is cleaved at the C-terminal. (A) Full-length recombinant human tau protein (rTau4R) was still detectable in the culture medium after being added to control Hela cells for 48 hrs (arrow, M+cells). rTau4R was less degraded when it was added to M without cells (M) (B) Secreted tau is not cleaved at the N-terminal as revealed by the anti-tau antibody Tau12 directed against the 9–18 a.a. A Tau12-positive band was detected in both L and M prepared from Hela cells overexpressing GFP-tau4R corresponding to full-length and cleaved tau (upper and lower arrows). (C) GFP tag inserted at the N-terminal of tau was detected in tau present in both L and M (upper and lower arrows). (D) The microtubule-binding domain of tau was not cleaved in tau secreted by Hela cells as revealed by the K9JA antibody (lower arrow). (E) The band found at 75 kDa in both L and M was not detected by the antibody Tau46 that recognizes the peptidic sequence located between L428 and L441. Only full-length tau present in L was detected with this antibody (arrow). A non-specific band at ∼100 kDa was noted with the antibody Tau46 in M. The pattern of each antibody was analyzed at least in 3 sets of experiments.

Tau secreted in the culture medium was consistently cleaved. Two observations pointed out that tau was most likely cleaved before being secreted by Hela cells. First, the tau-positive band at 75 kDa as well as lower tau-positive bands present in the culture medium were often observed in the cell lysate. Second, when recombinant human tau protein was added to the culture medium of control cells for 48 hrs, full-length tau was detectable although some degradation had occurred (Figure 4A). This indicated that if full-length tau was secreted by Hela cells, it should not have been completely degraded in the culture medium after 48 hrs of transfection.

A panoply of antibodies directed against different regions of tau were employed to analyze the cleavage pattern of tau in the culture medium and cell lysate (Table 1). In the cell lysate, all antibodies tested could detect full-length tau (Figure 4B, C, D and E). The band found at 75 kDa in both the cell lysate and culture medium was also detected by all the antibodies tested except for the antibody Tau46 that recognizes the peptidic sequence located between L428 and L441 [38]. This indicated that tau found at 75 kDa was cleaved at the C-terminal as reported for tau present in the CSF of both humans and tau transgenic mice and for tau secreted by M1C and NB2a/d1 cells [9], [14], [27], [28], [29], [30].

The band located at 75 kDa mainly present in the medium was immunoreactive to the antibody Tau12 directed against an epitope (9–18 a.a.) located at the N-terminal of tau (Figure 4B). This band was also detected by an anti-GFP antibody confirming that no cleavage had occurred at the N-terminal of tau where the GFP tag was inserted (Figure 4C). The antibody Tau12 could also detect bands found between 37 and 50 kDa and bands between 25 and 37 kDa revealing that the N-terminal was contained in these tau truncated forms (Figure 4B).

The antibody K9JA directed against an epitope located in the microtubule-binding domain (MTBD) of tau revealed the band found at 75 kDa and bands between 37 and 50 kDa and bands between 25 and 37 kDa (Figure 4D). At around 37 kDa, a strong signal was detected with the anti-tau antibody Tau12 and the anti-GFP antibody whereas a weak signal was noted with the K9JA antibody indicating that the MBTD could be cleaved in these tau fragments. Finally, no band lower than 75 kDa was detected with the antibody Tau46 directed against an epitope located at the C-terminal. This implies that the lower tau fragments were generated from cleaved tau found at 75 kDa lacking the C-terminal in both the cell lysate and culture medium (Figure 4E).

### Distinct Phosphorylation Pattern of Intracellular and Extracellular Tau

**Figure 5:**
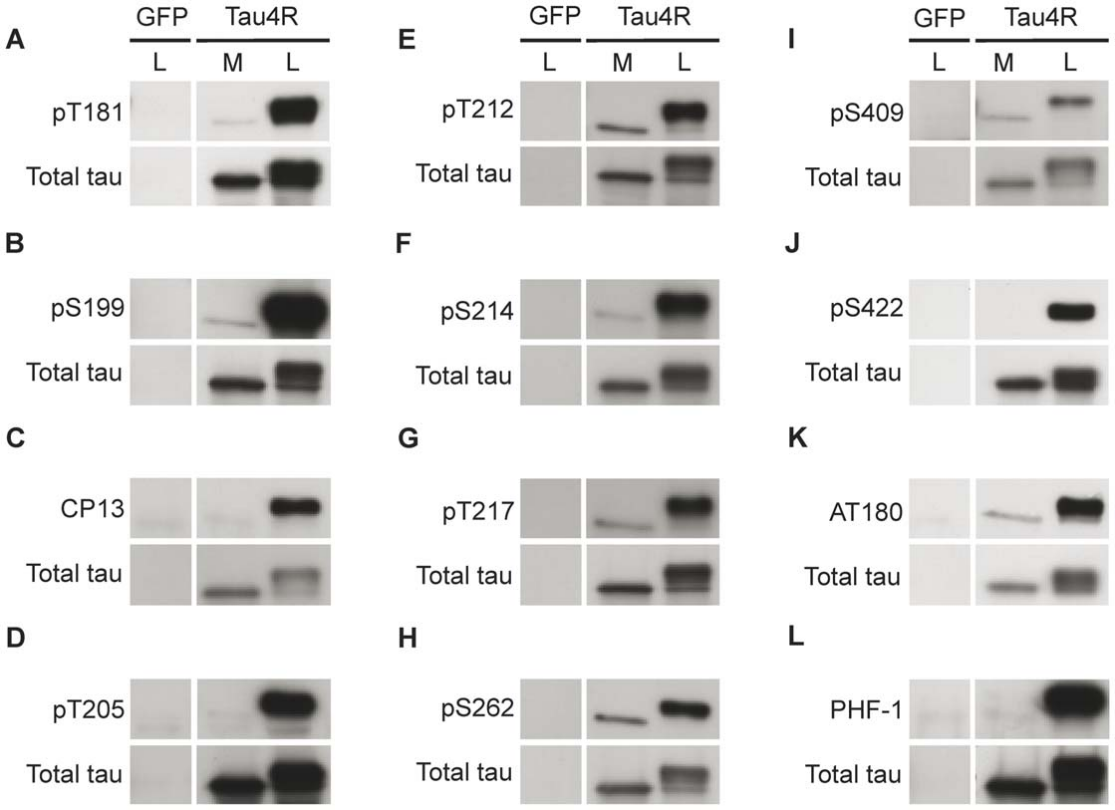
Secreted tau is dephosphorylated compared to intracellular tau. (A, B, E, F, G, H, I and K) Secreted tau was phosphorylated at T181, S199, T212, S214, T217, S262, S409 and at the epitope of the AT180 antibody (T231/S235) but to a lesser extent than intracellular tau. (C, D, J and L) No signal was detected in M with the phospho-tau antibodies directed against phosphorylated S202 (CP13), T205 (pT205), S422 (pS422) and the S396/S404 (PHF-1) whereas a strong signal was observed in L with these antibodies. Tau12 antibody was used to reveal total tau in M and L. The pattern of each antibody was analyzed at least in 3 sets of experiments.

In the cell lysate, full-length tau was phosphorylated at several sites known to be hyperphosphorylated in Alzheimer brain including early (T181, AT8 and S262), intermediate (AT100 and AT180) and late sites (PHF-1 and S422) (Figure 5). Surprisingly, several sites phosphorylated in intracellular tau were either not phosphorylated or less importantly phosphorylated in extracellular tau. The phosphorylation of two of the three sites (S199, S202 and T205) forming the epitope of the antibody AT8, S202 and T205, was not detectable in the medium although a strong signal was observed in the cell lysate (Figure 5C and D). A similar observation was made for the PHF-1 antibody recognizing tau phosphorylated at S396 and S404 as well as for the antibody pS422 directed against tau phosphorylated at S422 (Figure 5J and L). Extracellular tau was phosphorylated at the sites contained in the epitope of the antibodies AT100 (T212/S214/T217) and AT180 (T231/S235) as well as T181, S199, S262 and S409 (Figure 5A, B, E, F, G, H, I and K). For most phospho-tau antibodies, a band was detected in the medium only after a long film exposure which resulted in the saturation of the signal observed in the cell lysate. This indicated that tau found in the medium was less phosphorylated than intracellular tau. To confirm this, the amount of total tau in the medium and cell lysate was examined using the phospho-independent antibody Tau12. As illustrated in Figure 5, the amount of total tau and phospho-tau was similar in the cell lysate whereas in the medium, the amount of total tau was significantly higher than that of phospho-tau. The above results led us to conclude that extracellular tau was significantly less phosphorylated than intracellular tau.

### Phosphorylation Favors the Secretion of Tau by Hela Cells

**Figure 6:**
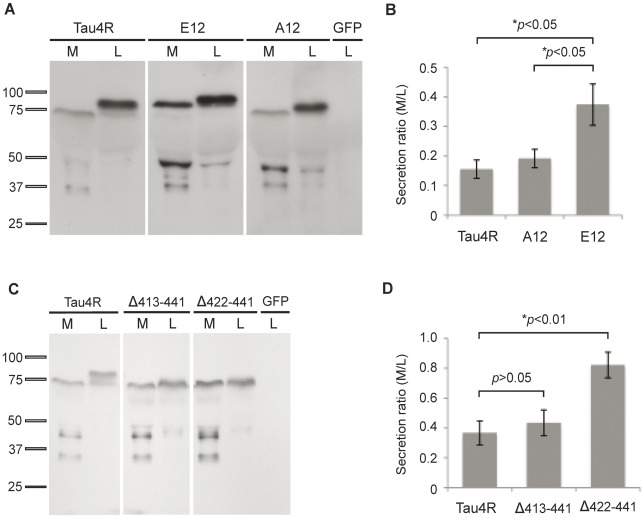
Phosphorylation and cleavage at the caspase-3 site enhance tau secretion by Hela cells. (A) Secreted A12 and E12 mutants were cleaved in M as their migration was faster in M than in L. A12 was secreted in a manner similar to that of wild-type tau whereas E12 was more secreted than wild-type tau and A12. In both M and L, E12 displayed a decrease in electrophoretic mobility compared to wild-type tau and A12. The Tau12 antibody was used to reveal tau in L and M. No signal was detected in L prepared from Hela cells transfected with the empty GFP vector (GFP). (B) Quantification of the secretion ratio M/L of wild-type tau (tau4R), A12 and E12. (C) Two tau mutants cleaved at either S412 (Δ413–441) or D421 (Δ422–441), the cleavage site of caspase-3, were secreted by Hela cells. TauΔ422–441 was significantly more secreted than wild-type tau whereas tauΔ413–441 was secreted at similar levels as wild-type tau. No signal was noted with the anti-tau antibody, Tau12, when cells were transfected with the empty GFP vector (GFP). (D) Quantification of the secretion ratio M/L of wild-type tau (tau4R), tauΔ413–441 and tauΔ422–441. The bars represent the mean of 4 experiments + SEM. *p*< 0.05.

The decreased phosphorylation of tau found at 75 kDa in the culture medium could indicate that either dephosphorylation favored the secretion of tau or dephosphorylation of tau occurred during the process of secretion in Hela cells. To verify whether dephosphorylation enhanced tau secretion, a tau mutant presenting mutations in alanine at 12 sites (A12) known to be phosphorylated in Hela cells was generated and overexpressed in these cells. Secreted A12 mutant was cleaved and was not more secreted than wild-type tau indicating that dephosphorylation would not be a determinant factor in tau secretion (Figure 6A). To further investigate how phosphorylation modulated tau secretion by Hela cells, we produced a mutant where the 12 above sites were mutated in glutamate (E12) to mimic phosphorylation. Interestingly, this mutant was more secreted than wild-type tau and A12 mutant (Figure 6A). E12 secreted by Hela cells was also cleaved as observed for wild-type tau and A12 mutant. To measure the secretion of wild-type tau, A12 and E12 mutants, the signal of the tau-positive band found at 75 kDa in the medium was quantified by densitometry as well as the signal of the band corresponding to full-length tau and that at 75 kDa present in the cell lysate. The secretion of tau was evaluated by calculating the ratio of the signal obtained with the anti-tau antibody Tau12 in the culture medium (M) and cell lysate (L) (Ratio M/L). The mean of the ratio M/L was 0.16±0.03, 0.37±0.070 and 0.19±0.03 for wild-type tau, E12 and A12 respectively (Figure 6B). The secretion of E12 was significantly higher (∼2 times) than that of wild-type tau and A12. The percentage of cell death for wild-type tau, E12, A12 and was 4.9±1.59, 4.33±1.95 and 2.57±1.67 respectively. From the above data, one could conclude that phosphorylation favored the secretion of tau by Hela cells.

### Tau Cleaved at D421 is Preferentially Secreted by Hela Cells

Tau present in the culture medium was always cleaved. This could signify that cleaved tau was preferentially targeted to the secretory pathway. To verify this possibility, tau mutants cleaved at the C-terminal were overexpressed in Hela cells. In the previous section (Figure 5), the staining of secreted tau with different phospho-dependent anti-tau antibodies indicated that tau could be cleaved between the a.a. S409 and S422. Indeed, truncated tau present in the medium was immunoreactive to the antibody pS409 but not to the antibody pS422 indicating that this latter site may be cleaved. Two mutants cleaved at either S412 (Δ413–441) or D421 (Δ422–441), the cleavage site of caspase-3, were produced and overexpressed in Hela cells [39]. Forty-eight hrs after transfection, the medium was collected and cells were lysed to analyze the presence of cleaved tau in the medium and cell lysate by western blotting. Both cleaved tau mutants were secreted by Hela cells (Figure 6C). Interestingly, tauΔ422–441 was significantly more secreted than wild-type tau whereas tauΔ413–441 was secreted at levels similar to wild-type tau. This was well illustrated by the fact that the tauΔ422–441 mutant was more abundant in the culture medium than in the cell lysate, a distribution that was never observed with wild-type tau. To measure the secretion of wild-type tau, tauΔ413–441 and tauΔ422–441, the signal of the tau-positive band found at 75 kDa in the medium and the signal of the band corresponding to full-length tau and that at 75 kDa present in the cell lysate were quantified by densitometry. The secretion of tau was evaluated by calculating the ratio of the signal obtained with the anti-tau antibody Tau12 in the culture medium and cell lysate (Ratio M/L) as described in the previous section. The mean of the ratio M/L was 0.37±0.08, 0.44±0.09 and 0.82±0.09 for wild-type tau, tauΔ413–441 and tauΔ422–441 respectively (Figure 6D). For both tauΔ413–441 and tauΔ422–441, it appeared that they were either not cleaved or only cleaved of few a.a. at the C-terminal during the process of secretion since the highest tau-positive band presented a similar molecular weight in both the cell lysate and culture medium. All together, these results revealed that cleavage of tau at the C-terminal was a crucial step for its secretion and that the cleavage site was a determinant factor regulating the amount of tau that was secreted by Hela cells.

**Figure 7:**
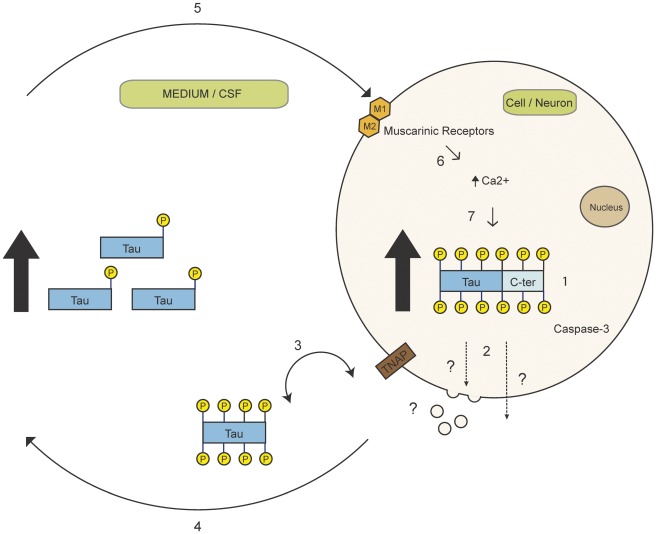
A schematic representation of the vicious cycle leading to the amplification of tau secretion in AD. In AD, tau becomes hyperphosphorylated (1). This hyperphosphorylation would enhance its secretion by either exosomes/microvesicles or another unconventional secretory pathway (2). Extracellular hyperphosphorylated tau would be dephosphorylated by TNAP present at the plasma membrane (3) and this would result in an increase of dephosphorylated tau in the extracellular space (4). Dephosphorylated extracellular tau would activate the muscarinic receptors (5) and this would induce an increase of intracellular calcium (6), an event linked to the increase of tau hyperphosphorylation (7). This further increase of hyperphosphorylated tau would initiate a vicious circle that would enhance tau secretion.

Since tau is preferentially cleaved at D421 by caspase-3, the activity of caspase-3 was inhibited by Z-DEVD-FMK in Hela cells overexpressing wild-type tau [39]. The secretion of tau was evaluated by calculating the ratio of the signal obtained with the anti-tau antibody Tau12 in the culture medium and cell lysate (Ratio M/L) as described above. The mean of the ratio M/L was.64±0.076 and.42±0.062 for control and treated cells respectively. The ratio M/L of control cells was significantly higher than the ratio of cells treated with the caspase-3 inhibitor. However, the fact that the difference of tau secretion between control and treated cells was ∼20% indicated that only a small pool of secreted wild-type tau was cleaved by caspase-3. This is consistent with the fact that wild-type tau was less secreted than the form of tau truncated at D421.

## Discussion

In the present study, we demonstrated that overexpressed human tau was secreted by Hela cells through an unconventional secretory pathway. The pool of secreted tau was cleaved at the C-terminal and was less phosphorylated than intracellular tau. Both hyperphosphorylation and cleavage at D421 enhanced tau secretion by Hela cells.

Our results demonstrating that tau was secreted by an unconventional secretory pathway is consistent with recent studies reporting that tau was found in exosomes in culture medium from MC1 cells overexpressing human tau and that secreted tau by COS-7 and HEK-293 cells was present in microvesicles [11], [30]. It remains to be determined whether tau utilizes other non-conventional pathways since a portion of tau in the culture medium of MC1 cells was not associated with exosomes. In the present study, tau secreted by Hela cells could be immunoprecipitated from the culture medium without using any detergent indicating that it was not included in microvesicles/exosomes. Consistent with this, no decrease of tau in the medium was observed after the culture medium was deprived of microvesicles by ultracentrifugation. All together the above results indicate that tau is most likely secreted by more than one pathways as shown for other proteins involved in neurodegenerative diseases such as SOD1 associated with Amyotrophic lateral sclerosis and the prion protein [40], [41], [42], [43]. In our previous study, we showed that hyperphosphorylated tau was preferentially associated with the rough endoplasmic reticulum (RER) membranes in AD brain and in the tau transgenic mice JNPL3 [44]. An increase of hyperphosphorylated tau was also noted at the Golgi apparatus in the JNPL3 mice [44]. RER and Golgi have been showed to be involved in non-conventional secretory pathways [45]. For example, COPII vesicles budding from the ER and containing tau at their surface could directly fuse with the plasma membrane for secretion [45]. This pathway is also used by the signal-peptide-containing protein, cystic fibrosis transmembrane conductance regulator (CFTR) [46]. Another possibility is that tau secretion could occur through non-COPII-coated vesicles forming at the ER or vesicles forming at the Golgi having tau attached at their surface [47]. We reported that Tau was found at the surface of RER membranes but this does not exclude the possibility that it could end up on the extracellular surface of the plasma membrane during the fusion process occurring between tau-containing vesicles and the plasma membrane.

Our results demonstrated that cleavage of tau at D421 increased its secretion. The fact that wild-type tau and tauΔ413–441 were secreted in a similar way by Hela cells strongly suggests that the major pool of secreted wild-type tau could be cleaved close to S412 in Hela cells. To further demonstrate this, Hela cells overexpressing wild-type tau were treated with a caspase-3 inhibitor since tau is preferentially cleaved at D421 by this caspase [39]. When Hela cells were treated with a caspase-3 inhibitor, a small but significant decrease of wild-type tau secretion was observed. This could indicate that as mentioned above, the major pool of wild-type tau secreted by Hela cells was not cleaved at D421. From our results, it was not possible to conclude whether tauΔ413–441 and tauΔ422–441 underwent further cleavage during the process of secretion. The fact that they migrated in a similar way in the cell lysate and culture medium could signify that if they were cleaved it was only by a few amino acids. The secretion of tau cleaved mutants indicates that wild-type tau was most likely cleaved before its trafficking in the secretory pathway. Based on a recent study reporting that an increase of caspase activity is an early event in AD and our results showing the enhanced secretion of tau cleaved at the caspase-3 site, one can speculate that the secretion of tau would be enhanced at the initial stage of the disease [48].

Mimicking of hyperphosphorylation significantly enhanced the secretion of tau by Hela cells. However, tau found in the culture medium was dephosphorylated compared to the pool of tau that remained intracellular. This is consistent with a recent study reporting that released tau in the culture medium upon cell lysis was dephosphorylated compared to intracellular tau [26]. In this study, extracellular tau was not phosphorylated at the epitopes recognized by the phospho-tau antibodies, AT8 and PHF-1 whereas intracellular tau was. Tissue non-specific alkaline phosphatases (TNAP) present in the plasma membrane were shown to be responsible for the dephosphorylation of extracellular tau [26]. Interestingly, TNAP were shown to be increased in AD brain [26]. All together, the above data indicate that CSF-tau might be less phosphorylated than intracellular tau. Several studies have examined the phosphorylation of tau found in the CSF of patients affected by a tauopathy. T181 and T231 are the sites that have been extensively used as a diagnostic tool for AD [8]. Although in most studies, the phosphorylation of T231 was shown to be increased in AD, some studies reported that its phosphorylation was reduced at later stages of the disease [18]. However, the phosphorylation of other sites such as the epitopes of AT8 and PHF-1 remains controversial [8]. Our data revealed that these epitopes are preferentially dephosphorylated in secreted tau. The AT8 epitope seems to play a central role in the hyperphosphorylation cascade of tau. Indeed, an increase in phosphorylation of the AT8 epitope is detected at an early stage of AD [49], [50], [51]. In our previous study, we reported that the phosphorylation of the AT8 epitope had the most significant effects on the phosphorylation of other sites in primary hippocampal neurons [52]. The results of the present study highlight the possibility that the dephosphorylation of this epitope could be regulated in a distinct manner.

In a previous study, it was shown that dephosphorylated tau in the culture medium could act as an agonist of muscarinic M1 and M3 receptors inducing a robust and sustained increase of intracellular calcium that triggered cell death in SH-SY5Y cells [26]. Most importantly, the increase in intracellular calcium induced by dephosphorylated tau in the culture medium was associated with an increase of TNAP expression [26]. Based on these observations and our present data, one could speculate that tau found in the extracellular space in AD brain would be dephosphorylated and thereby would contribute to the aberrant homeostasis of calcium noted in this tauopathy.

From our data and that of other groups, it appears that both extracellular and intracellular tau could contribute to the process of neurodegeneration linked to AD. Furthermore, our data indicate that in AD, hyperphosphorylation of tau would induce a vicious circle that would result in the amplification of its secretion (Figure 7). Indeed, our data revealed that hyperphosphorylation of tau would enhance its secretion and this would in turn increase the amount of dephosphorylated tau in the extracellular space. Dephosphorylated extracellular tau would then induce an increase of intracellular calcium, an event linked to the increase of tau hyperphosphorylation [26]. This increased hyperphosphorylation of tau would further enhance its secretion leading to the emergence of a vicious circle that would promote the propagation of tau pathology in the brain and its accumulation in the CSF. The accumulation of total and phospho-tau in the CSF is used as a diagnostic biomarker for tauopathies [8]. Our data highlight the possibility that the distinct phosphorylation and cleavage pattern of tau could account for its differential accumulation in the CSF among the tauopathies. The characterization of this pattern for each tauopathy could become a powerful tool for their early detection and to distinguish them from one another.
